# Monumental landscapes of the Holocene humid period in Northern Arabia: The mustatil phenomenon

**DOI:** 10.1177/0959683620950449

**Published:** 2020-08-17

**Authors:** Huw S Groucutt, Paul S Breeze, Maria Guagnin, Mathew Stewart, Nick Drake, Ceri Shipton, Badr Zahrani, Abdulaziz Al Omarfi, Abdullah M Alsharekh, Michael D Petraglia

**Affiliations:** 1 Extreme Events Research Group, Max Planck Institute for Chemical Ecology, Jena, Germany; 2 Department of Archaeology, Max Planck Institute for the Science of Human History, Jena, Germany; 3 Max Planck Institute for Biogeochemistry, Jena, Germany; 4 Department of Geography, Kings College London, London, UK; 5 Centre of Excellence for Australian Biodiversity and Heritage, College of Asia and the Pacific, Australian National University, Australia; 6 Institute of Archaeology, University College London, UK; 7 Ministry of Tourism of Saudi Arabia, Riyadh, Saudi Arabia; 8 Department of Archaeology, College of Tourism and Archaeology, King Saudi University, Riyadh, Saudi Arabia; 9 Human Origins Program, Smithsonian Institution, Washington, DC, USA; 10 School of Social Science, The University of Queensland, Brisbane, Australia

**Keywords:** archaeology, climate change, human-environment interaction, Neolithic, pastoralism, territoriality

## Abstract

Between 10 and six thousand years ago the Arabian Peninsula saw the most recent of the ‘Green Arabia’ periods, when increased rainfall transformed this generally arid region. The transition to the Neolithic in Arabia occurred during this period of climatic amelioration. Various forms of stone structures are abundant in northern Arabia, and it has been speculated that some of these dated to the Neolithic, but there has been little research on their character and chronology. Here we report a study of 104 ‘mustatil’ stone structures from the southern margins of the Nefud Desert in northern Arabia. We provide the first chronometric age estimate for this type of structure – a radiocarbon date of ca. 5000 BC – and describe their landscape positions, architecture and associated material culture and faunal remains. The structure we have dated is the oldest large-scale stone structure known from the Arabian Peninsula. The mustatil phenomenon represents a remarkable development of monumental architecture, as hundreds of these structures were built in northwest Arabia. This ‘monumental landscape’ represents one of the earliest large-scale forms of monumental stone structure construction anywhere in the world. Further research is needed to understand the function of these structures, but we hypothesise that they were related to rituals in the context of the adoption of pastoralism and resulting territoriality in the challenging environments of northern Arabia.

## Introduction

### The early to middle Holocene archaeology and climate of Arabia

The study of the prehistory of Arabia has lagged behind other regions, but recent advances have begun to correct this imbalance (e.g. Groucutt and Petraglia, 2012; Magee, 2014; Petraglia and Rose, 2009; Petraglia et al., 2015). Human occupations began by at least the late Middle Pleistocene (e.g. Scerri et al., 2018a), and are increasingly well understood in the Late Pleistocene (e.g. Armitage et al., 2011; Delagnes et al., 2012; Groucutt et al., 2015, 2018). Holocene archaeological sites are much more abundant than those of the Pleistocene (e.g. Drechsler, 2009; Guagnin et al., 2017a, 2020; Magee, 2014; Petraglia et al., 2020; Scerri et al., 2018b; Zielhofer et al., 2018). Most research has focussed on the southeast of Arabia, and little remains known for vast areas of the peninsula. Recent discussions on topics such as the ‘Neolithization’ process (Crassard and Dreschsler, 2013) have contrasted a focus on indigenous/autochthonous developments (e.g. Crassard and Khalidi, 2017) and an emphasis on migration into the area, bringing in changes such as animal domestication (e.g. Uerpmann et al., 2009).

One fascinating aspect of the Holocene archaeological record of Arabia concerns the abundant stone structures which were constructed across the area. As well as being a widespread and highly visible part of the archaeological record, they provide an independent perspective on processes of demographic and cultural change compared to other datasets such as stone tools. Here we explore the origin and development of Arabian stone structures in the context of the wider environmental and archaeological records. Cairns – typically relatively small structures with a funerary function (e.g. Abu-Azizeh et al., 2014; Alsharekh, 2006; Guagnin et al., 2020; Harrower et al., 2013) – are found across the region and occurred from the Neolithic to at least ca. 600 AD (Alsharekh, 2004). As discussed more below, a recently published stone platform from Dûmat al-Jandal also had a funerary aspect, and dates to the sixth millennium BC (Munoz et al., 2020). Little is known of larger, more complex, forms of stone structure. Of particular interest for earlier periods are forms known as desert kites and, as focussed on in this paper, mustatils which have previously been described as ‘gates’ (Kennedy, 2017). Desert kites are generally seen as mass-kill hunting traps, and although common in northern Arabia, no detailed work has yet been conducted on them in the peninsula, so their chronology remains unclear (Crassard et al., 2015; Kennedy et al., 2015). Further north, in Jordan, as discussed below, a single desert kite was recently dated to an estimated ca. 8000 BC (Al Khasawneh et al., 2019), indicating the considerable antiquity of large-scale stone structures. In Arabia, desert kites and particularly mustatils have been argued to be the oldest forms of stone structure in the landscape, as they consistently underlie later forms of stone structure (e.g. Guagnin et al., 2020; Kennedy, 2017). However, the precise age of these older forms, their function, and origins are all currently unclear.

In southern Arabia, the oldest monumental stone structures consist of stone platforms (McCorriston et al., 2011, 2012). For instance, in Yemen’s Wadi Sana, McCorriston and colleagues (2012) identified a least 40 stone platforms. Most have not been studied in detail, but many are associated with medium to large animal bones, leading McCorriston and colleagues to argue they were locations where animals were sacrificed in the context of increased territoriality. Shi’b Kheshiya is a particularly spectacular site, where a ring of 42 skulls of domesticated adult cattle were carefully arranged in a circle, in what appears to be a single event around 4400 BC (McCorriston et al., 2012). Shi’b Kheshiya is interpreted as indicating feasting, in which people and animals from a large area converged in Wadi Sana (e.g. Henton et al., 2014; McCorriston and Martin, 2009).

Indications of the social and economic context in which large-scale monumentalism emerged in Arabia come from sites in southern and eastern Arabia indicating the presence of pastoralism perhaps as early as ca. 6800 BC, and more strongly by ca. 6000 BC (e.g. Drechsler, 2007, 2009; Martin et al., 2009; McCorriston and Martin, 2009; Uerpmann et al., 2009, 2013). The Neolithic took on different forms in different regions, and in the challenging and often arid environments of Arabia, pastoralism and hunting were not exclusive options, but were instead often combined (e.g. McCorriston, 2013; McCorriston and Martin, 2009). As well as the findings from Shi’b Kheshiya mentioned above, changing social dynamics are visible in features such as the appearance of cemeteries and the construction of elaborate cairns, both of which have been argued to indicate increased attachment to particular places in the landscape and growing territoriality (e.g. Harrower, 2008; Magee, 2014). At Jebel Buhais, hundreds of people were buried in the cemetery between ca. 5200 and 4000 BC, sometimes with elaborate grave goods, and many bear marks of violent deaths (Kiesewetter, 2006). This evidence presumably reflects a combination of social changes and responses to environmental fluctuation, such as the transition to aridity at the end of the Holocene humid period (e.g. Petraglia et al., 2020).

Our research programme in northern Arabia has been exploring long-term cultural and environmental changes, and addresses themes such as how the Arabian and Levantine records relate. The sites of Jebel Oraf 2 (ca. 5300 to 4300 BC) and Alshabah (ca. 5300 to 4500 BC) in the Nefud Desert have been interpreted as pastoral sites (Guagnin et al., 2017a, 2020; Scerri et al., 2018b). Lithic assemblages from northern Arabia provide indications of connections with the Levant (e.g. Crassard et al., 2013; Crassard and Hilbert, 2019; Guagnin et al., 2020; Hilbert et al., 2014). Unlike virtually all Neolithic sites in the Levant, Alshabah and Jebel Oraf 2 lack structural remains of dwellings. It is likely that these groups utilised high mobility as a way to ensure survival in a region where droughts were probably common even during the Holocene humid period (Guagnin et al., 2016). Prolific aquifers, a high groundwater table, and the ability to move to different areas which had received rainfall probably provided the means for these groups to survive conditions which were probably challenging and variable even during the wettest part of the Holocene humid period (Petraglia et al., 2020).

Contextualising the above social changes, recent studies have provided considerable climatic and environmental detail, particularly in southeast Arabia (e.g. Preston and Parker, 2013; Preston et al., 2015). The Holocene humid Period, broadly 8000–4000 BC, saw a significant increase in precipitation in Arabia. This led to the formation of lakes and other wetlands (e.g. Engel et al., 2017), the activation of rivers (Matter et al., 2016), speleothem formation (Fleitmann et al., 2007, 2011), and major changes in vegetation (Dinies et al., 2015). Records from the early to middle Holocene from northern Arabia remain patchy (Crassard et al., 2013; Dinies et al., 2015; Engel et al., 2012; Hilbert et al., 2014; Schulz and Whitney, 1986; Whitney et al., 1983). The only long and relatively continuous record of early to middle Holocene environmental dynamics in northern Arabia come from Tayma. Increased rainfall in the Tayma area led to the spread of grasslands, peaking between ca. 6600 and 6000 BC, after which there was a return to arid adapted vegetation (Dinies et al., 2015). The palynological data matches geoarchaeological and palaeohydrological data from Tayma for a decline in lake levels from ca. 6000 BC (Engel et al., 2012). Indications from elsewhere in the area, such as at the Jubbah oasis, suggest that there was continued water availability in some locations after 6000 BC (e.g. Crassard et al., 2013; Hilbert et al., 2014). Indeed, at Jebel Oraf the highest lake stand occurred around 5300 BC (Guagnin et al., 2020). Given the spatio-temporal complexity and time-transgressive pattern of the end of humid conditions known from similar latitudes in the Sahara (Shanahan et al., 2015), and the extensive recharge of aquifers in northern Arabia during peak early Holocene humidity, it is not currently clear how the landscape changed in the later part of the Holocene humid period in different areas of northern Arabia.

In Arabia, as discussed above, evidence for the origin of pastoralism and the construction of the oldest known stone structures (simple cairns and the Dûmat al-Jandal platform) correlate with the wet conditions of the Holocene humid period, while in southern and eastern Arabia, the emergence of increasingly territorial behaviours have been correlated with the end of the humid period. Understanding the process between these points, both spatially and temporally, represents a key avenue for research in Arabia. As well as understanding broad intra- and inter-regional developments in the Holocene, further studies are needed to explore the background to the development of oasis agriculture (see e.g. Hausleiter et al., 2018). Given the earlier end of the Holocene humid period in northern compared to southern Arabia (e.g. Fleitmann et al., 2007) we would predict an earlier expression of territorial behaviours in the north. However, given a lack of knowledge on how climatic changes led to environmental changes, the precise character of changing water availability remains an important topic for future research.

### Stone structures and the mustatil phenomenon

**Figure 1.**
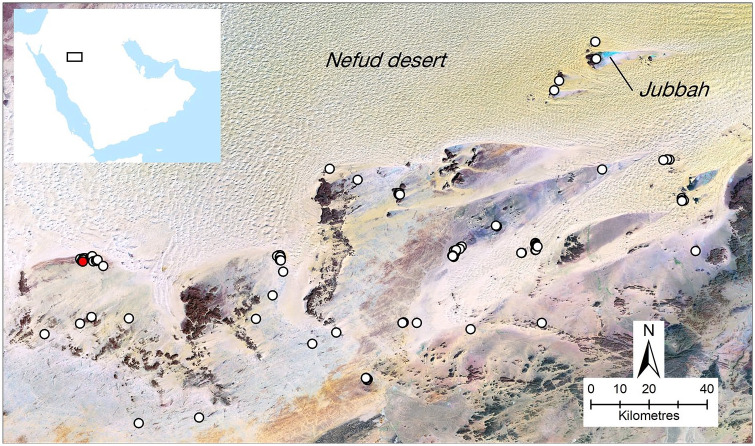
The distribution of mustatils in the study area, the southern margins of the Nefud Desert. The red dot shows the location of the dated mustatils (see Figure 4 for detail).

**Figure 2.**
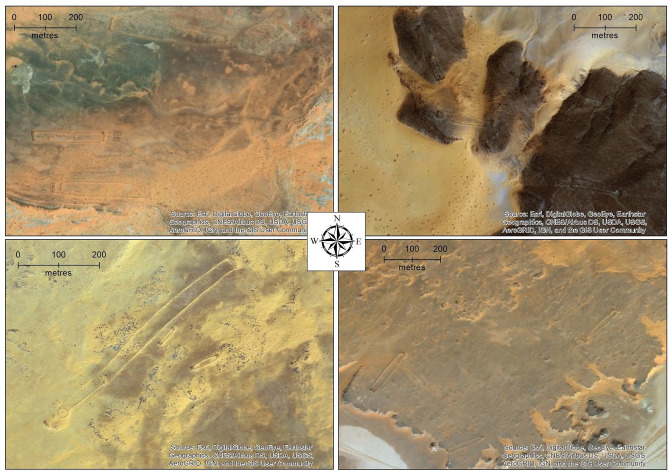
Examples of groups of mustatils in the southern Nefud. They appear as faint rectangular shapes. Note variable landscape positions and orientations, and frequent evidence for later alteration. Clockwise from top left: (1) three parallel east-west orientated mustatils, (2) a broadly linear arrangement of four mustatils along jebels, (3) five mustatils from just north of a playa in the area shown in Figure 4, (4) two small and one large mustatils from the area shown in Figure 4. The large mustatil is the largest such structure recorded anywhere in Arabia. The southwestern end of this mustatil has been re-used to build both a keyhole and a pendant, among other structures.

**Figure 3.**
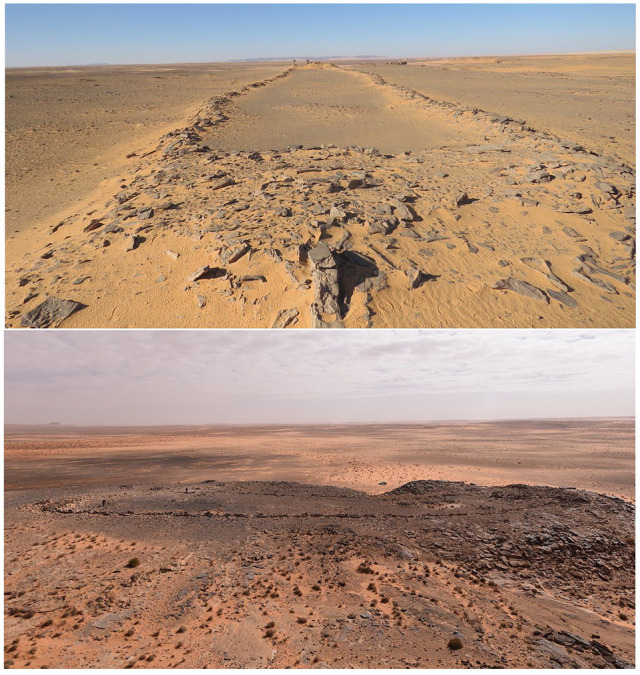
Two examples of mustatils in the study area. Note the platform in the foreground of the top image (from Figure 4 area), taken along the axis of a mustatil. The image at the bottom shows a side-on view of the Jebel Dhaya mustatil near Jubbah, the paired platforms at either end of the mustatils are visible. Scale in both images is provided by team members.

While few excavations and other detailed investigations of early to middle Holocene archaeological sites have been conducted in northern Arabia, several studies have used remote sensing approaches to explore the human past of the area. This has taken the form of mapping and analysis of the various sorts of stone structures which are abundant in northwest Arabia, as well as in the adjacent southern Levant (e.g. Abu-Azizeh et al., 2014, 2015; Crassard et al., 2015; Kennedy, 2011, 2017; Kennedy and Al-Saeed, 2009). Kennedy and Al-Saeed (2009) described one enigmatic form of stone structure that they called ‘gates’, due to their resemblance to farm gates when viewed from satellite imagery. As we show in this paper, these structures are more widespread than previously known (Figure 1; for list of sites reported here see: supplementary data table). Figures 2 and 3 show how examples of these structures look from above and from the ground respectively.

Based primarily on the analysis of satellite imagery, Kennedy (2017: 156) gives the only previous detailed study of these structures, which he defined as ‘two short, thick lines of heaped stones, roughly parallel, linked by two or more much longer and thinner walls’. In the Harrat Khaybar area, they typically have two long walls, but sometimes three or even four parallel walls (hence the ‘gate’ like appearance). As we will discuss below, in our study area there are rarely multiple long parallel walls, with almost all examples only having two, so the name ‘gate’ does not seem particularly fitting. Likewise, our on the ground study emphasises that where not disturbed by later activity, these structures are characterised by flat-topped stone platforms at either end. These platforms are not clearly visible in satellite imagery, creating the impression of ‘two short thick lines of heaped stones’. These structures are termed ‘mustatil’, which is Arabic for ‘rectangle’, a term created by archaeological teams working under the auspices of the Royal Commission for AlUla. Our focus here is on describing the mustatils of the southern Nefud, which can be defined as stone structures where two stone platforms, roughly parallel in long-axis orientation, are connected into an elongate rectangular shape by parallel long and thin walls connecting the short-axis ends of the platforms. Future work will add more nuance to this basic definition.

Kennedy (2017) focussed on the Harrat Khaybar area, although noting that mustatils were also found further north and south of this area. In terms of their landscape positioning in Harrat Kaybar, mustatils are often located close to wadis (ephemeral river channels) flowing from the lava-field and claypans which would hold water after heavy rainfall (Kennedy, 2017). This is not always the case, however, as the group on the flanks of a remote volcano (25.678048 N, 39.964294 E) noted by Kennedy demonstrates. Kennedy (2017) emphasises that in relative chronological terms, mustatils are the oldest type of stone structure in the Harrat Khaybar area. This can be seen in the frequent re-use of stone from mustatils for other types of structure, but never the reverse. Kennedy therefore argues that this relative chronology suggests that mustatils could be as old as the Bronze Age or Neolithic.

While ‘on the ground’ research on mustatils is still in its infancy, studies of other types of stone structures provide important context. The luminescence dating of a desert kite close to the Saudi border in Jordan to ca. 8000 BC (Al Khasawneh et al., 2019), and of ‘wheels’ to seemingly Neolithic to Bronze age times (Athanassas et al., 2015; Rollefson et al., 2016), indicate that large-scale stone structures were being constructed on the margins of northern Arabia in the early to middle Holocene. Likewise, cairns, date from the Neolithic to at least the Iron Age (Abu-Azizeh et al., 2014; Guagnin et al. 2017b, 2020). In southern Jordan, cairns associated with platforms and other rectangular structures arranged in specific spatial arrangements were dated to the Neolithic periods, with the oldest cairn dating to 5341–5049 BC (Abu-Azizeh et al., 2014; see also Fujii, 2013). Likewise, three cairns at Jubbah have been dated to 6372–6060 BC, 5306–5216 BC and 5301–5061 BC respectively (Guagnin et al., 2017b, 2020). A recently published stone platform from Dûmat al-Jandal in northern Arabia (Munoz et al. 2020), on the other side of the Nefud Desert from our study area, is also interesting from the perspective of mustatils. This platform was constructed over several phases, beginning with a trapezoidal structure 20.6 m in length constructed around 5500–5600 BC. This structure included a mortuary aspect, and the platform was used and reconfigured over the following millennia. In southern Arabia, the earliest dated stone structures date to later periods, with platform structures at Shi’b Kheshiya dated to ca. 4400 BC (McCorriston et al., 2011). We will return to aspects of inter-regional comparison in the discussion at the end of this paper.

## Methods

The present study focuses on the southern margins of the Nefud Desert, where our interdisciplinary team has been researching the prehistory and palaeoenvironment of the region for the last decade. The study area for this paper is defined as the southern part of the Nefud Desert, from the western edge of Jebel Aja near Ha’il, to the ‘15’ road to Tayma in the west, and north of the ‘70’ road (Figure 1).

We carried out detailed inspection of high-resolution satellite data to identify the distribution of mustatils in the study area. Bing Maps imagery, and to a lesser extent, Google Earth, were used. As well as recording the location of each site, we recorded evidence of stratigraphic super-imposition (younger structures re-using stone from mustatils). GIS morphometric and spatial analyses were performed on the recorded features. Mustatil length, width, elongation (length/average width) and area (measured polygon), mean elevation above sea level (Supplemental Figure S3, available online), and relative orientation (Supplemental Figure S9, available online) were calculated using the spatial analysis tools in ESRI ArcGIS 10.5. Distance between mustatils and local water sources were calculated using the Near tool, based on the results of regional palaeohydrological mapping (Breeze et al., 2015), for all local water sources (lake, wetlands and drainage systems), and also considering only lakes/wetlands where water residence may have been longer (Supplemental Figures S4 and S5, available online).

**Figure 4.**
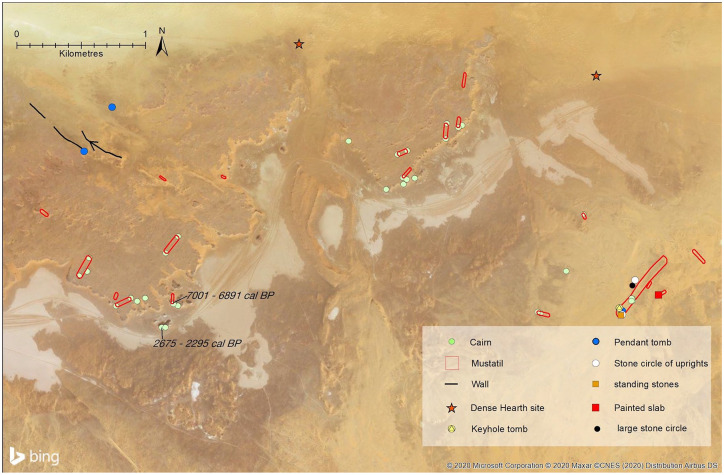
Case study area from southern Nefud with large number of mustatils and other kinds of stone structures. The edge of the Nefud sand sea is located at the northern edge of this area. Note distribution of mustatils along raised area just north of scarp above a series of playas (light coloured areas). A further group of mustatils and other structures occurs in the eastern part of this study area.

Having identified mustatils through the above analyses, we then carried out ground visits to selected sites. We explored localities in the area shown in Figure 4 in 2016, and those at Jubbah over several seasons. Sites were surveyed to elucidate their construction and associated material culture and photographs were taken. At one site, we found that persons unknown had dug into a mustatil, seemingly relatively recently, revealing an assemblage of animal bones and allowing us to recover a piece of charcoal from a section inside the platform of the mustatils. We also recovered a seashell from a cairn near the dated mustatil, which we dated to explore the chronology of the abundant cairns of the area. Samples were sent to Waikato Radiocarbon Dating Laboratory for dating. Sample WK45138 (seashell) was physically cleaned, then washed in an ultrasonic bath. It was then washed using 0.1N HCl, rinsed and dried. Sample WK45139 (charcoal) was physically cleaned, then washed in hot HCl, rinsed and treated with multiple hot NaOH washes. The NaOH insoluble fraction was treated with hot HCl, filtered, rinsed and dried. Both samples were measured with an AMS spectrometer. Results were then calibrated using OxCal (Bronk Ramsey, 2017; Supplemental Table S2, available online).

Faunal remains were identified to the lowest taxon possible through comparisons with photographs and relevant literature (e.g. Peters et al., 1997), although the poor preservation of most of the remains made species-level identification difficult. All measurements were taken following von den Driesch (1976) and each specimen was assessed for preservation and bone surface modifications following established methods (e.g. Behrensmeyer, 1978).

In the following sections, we first explore the distribution and landscape position of mustatils in the southern Nefud Desert, before focussing on a case study area in detail, which includes information on the chronology, associated finds, and morphological aspects. Finally, we evaluate the position of mustatils in the Holocene prehistory of Arabia and discuss their likely function.

## Results

### Distribution of mustatils

We identified a total of 104 mustatils around the southern margins of the Nefud Desert. This considerable number demonstrates that this structural form is also found in large numbers away from the Harrat Khaybar area emphasised by Kennedy (2017). We have not yet systematically evaluated other regions of Arabia for mustatils, however for the sake of future studies we note their continued presence to the east (see for example 27.9155 N, 42.2652 E, 27.9159 N, 42.2466 E and 27.9136 N, 42.2423 E). South of the Harrat Khaybar, mustatils appear to be rare, although structures positioned around 23 N in Harrat Kishb do seem to show a similar morphology (e.g. 23.0530 N, 41.0832 E). More common in western-central Arabia are smaller rectangular structures which may, or may not, prove to be related to the mustatil phenomenon. Examples of these include that at 23.006 N, 40.659 E; and three examples at 23.124 N, 40.470 E. Kennedy (2017) includes some of these more southerly forms in his list of mustatils, but they are small and square, instead of large and rectangular, and it is not clear if they are the same type of structure. Future fieldwork should evaluate these more southerly structures to see how they compare to those of Harrat Khaybar and the southern Nefud.

The distribution of mustatils in the southern Nefud Desert can be summarised by noting that, on the one hand, they are relatively evenly distributed across the study area (Figure 1), but that, on the other hand, they often occur as groups (Figures 2 and 4; Supplemental Figures S1 and S2, available online). This is illustrated by the median distance between mustatils being only 165.5 m (the minimum is 5 m, maximum 21,800 m), with 75% of mustatils being less than 681 m from their nearest neighbour. Thus, most of these structures were built close to others, concentrated in groups at specific points of the landscape, but with these groups fairly evenly distributed around the fringes of the southern Nefud. The way in which the mustatils are grouped varied; sometimes being broadly parallel in orientation, sometimes seemingly randomly distributed, and other times aligned in a linear fashion (e.g. Figure 2; see also linear example at 27.3997 N, 40.4824 E).

We explored the relationship of mustatils to various aspects of the landscape. In terms of altitude, mustatils in our study area occur between 851 and 1178 m above sea level. There is a particular concentration between 900 and 920 m above sea level (Supplemental Figure S3, available online). These values reflect the basic underlying topography of the area. While mustatils often seem to be built on slightly raised areas, they are not systematically located at overall topographic highs. There are some examples of mustatils built at high points, for instance on the jebels west of Jubbah (Figure 3), but these are exceptions, and even at Jubbah the largest mustatil is built at the base of, rather than atop, a jebel (sandstone hill). In this region elevated areas usually occur in the form of rocky outcrops that are a good source of the type of stones that were used as building materials. This illustrates the complexity in determining why mustatils were built in particular locations. Does their construction in an elevated position mean that the builders deliberately chose an elevated position, or because that is where a good supply of building stone occurred?

Likewise, landscape features are not independent in other regards. In the study area, palaeolakes and other wetlands are typically found in the lee of jebels, where a wind-shadow has minimised mass sand transport and dune migration, creating a relative topographic low. So being located at a high point and near a lake are not mutually exclusive. Many (approximately three-quarters) of the mustatils seem to be located on raised areas; but in many cases, these are also near to ancient lakes or wetlands. Furthermore, it is not always easy to classify landscape features in simple terms. A small eminence with vertical sides may create a more dramatic setting than somewhere with a higher altitude but a gradual slope. Future GIS studies will cast more light on these issues.

Some mustatils are found close to topographic lows and playas, which even today often hold water after heavy rainfall. Petraglia and colleagues (2020) highlight numerous such locations across northern Arabia as having episodically held surface water even under current conditions, based on a dataset of recent water occurrence (Pekel et al., 2016). In the wetter conditions of the early to mid-Holocene, many of these localities would have held ephemeral or seasonal lakes or wetlands. Examples of close associations between mustatils and these sources of surface water can be seen in Figure 4. This pattern is, however, not ubiquitous. Some mustatils seem to have been constructed away from any obvious water sources. Explored quantitatively, we found a median distance of 5596 m from mustatils to the nearest lake deposits (Supplemental Figure S4, available online). In terms of distance to any potential water source (i.e. including minor ephemeral streams, which may have only very infrequently held water), the median distance is 1,078 m (Supplemental Figure S5, available online). These figures indicate a *tendency*, while not an exclusive pattern, for mustatils to be located near water sources. The way in which mustatils relate to these water-bodies vary. For instance, in the area shown in Figure 4, many mustatils are arranged at approximately a right angle to the topographic lows. Here, many mustatils have a platform at one end close to the scarp overlooking the low areas. But even here, we can see that is not an exclusive pattern. Three small mustatils, for instance, are found several hundred metres north of the site we dated (Figure 4). Supplemental Figure S2, available online also illustrates a common landscape theme with mustatils in the study area: they are found near to, but slightly set back from, the lake basins they are sometimes found in proximity with. In summary, the high level of relative local-topographic complexity in the landscape inhibits identification of any definitive landscape pattern for the mustatils, should any exist. Clarity may come in the future by exploring the relationship between the position and chronology of different mustatils, and on better understandings of their function once excavations have been conducted.

### Size, shape and architecture

In terms of overall shape, the mustatils of the southern Nefud Desert are relatively homogenous. They are elongated rectangles, with 102 out of 104 examples having two long walls, and the other two having three. One of the examples with three walls is also the only clear example where the distal platforms extend outwards further than the point at which the long connecting walls join the platform. This is one of the two southern most mustatils in our study area (26.9005 N, 39.6969 E). These unusual features are relatively common in the Harrat Khaybar area (Kennedy, 2017) and may hint at patterns of regional variation in the morphology of mustatils.

In the southern Nefud Desert, the long walls are typically parallel, giving the mustatil an almost precisely elongate rectangular shape. In some cases, one or both long walls are not quite straight (e.g. 27.385731 N, 39.379182 E), occasionally causing a slight change of direction along the course of the mustatils (e.g. 27.386174 N, 39.377431 E).

Dimensional data on the southern Nefud mustatils are summarised in supplementary Table S1 and Figures 6–8, available online. They have a mean and median length of 161.1 and 142 m respectively, and while varying from 26 m to 616 m, between 100 m and 200 m in length is a typical size. The mean and median width is 21.7 m and 20.8 m respectively, with most cases falling between 15 and 30 m. Estimates of elongation and area were calculated to give insights into size. Elongation ratio (length/width) has a mean and median of 7.2 and 6.8 respectively, with most falling between 4 and 8, indicating a consistently elongated rectangular shape. Finally, area (m^2^) varies considerably, with mean and median values of 4363 m^2^ and 2950 m^2^ respectively, but high levels of variation giving areas from 259 m^2^ up to ~22,558 m^2^.The vast scale of these structures makes them among the most spectacular examples of prehistoric monumental architecture anywhere in the world. The mustatil located at 27.3865 N, 39.3780 E is the longest and largest so far recorded anywhere: 616 m in length and covering an area of more than 22,000 m^2^, with a large platform at either end. These southern Nefud structures – which seemingly occur on the margins of the broader distribution of mustatils, which appear to be concentrated in the Khaybar and Al Ula areas (Kennedy, 2017) – feature more than 30 km of walls in total, and contain thousands of tonnes of rocks, particularly in the platform ends.

In terms of the orientation of mustatils, there is no consistent overall pattern (supplementary Figure S9, available online). However, locally, there often does seem to be patterning, and mustatils in close proximity or distinct groups often share an overall orientation. For instance, in the area shown in Figure 4, most mustatils are orientated southwest/northeast (see also bottom two panels in Figure 2). However, in this example, there are several cases of smaller mustatils, positioned slightly away from the main groups, displaying a contrary northwest-southeast orientation. At the moment, there is no clear explanation for what this might mean, such as, whether it could represent diachronic change. Figure 2 shows the diversity of orientations: the top left panel, for instance, showing a group with east-west orientation. Bearing in mind the previous comment concerning close groupings, we can thus summarise the orientation of these mustatils as being regionally variable, but often locally homogenous. Supplementary Figures S1 and S2, available online show other examples of groups of mustatils.

**Figure 5.**
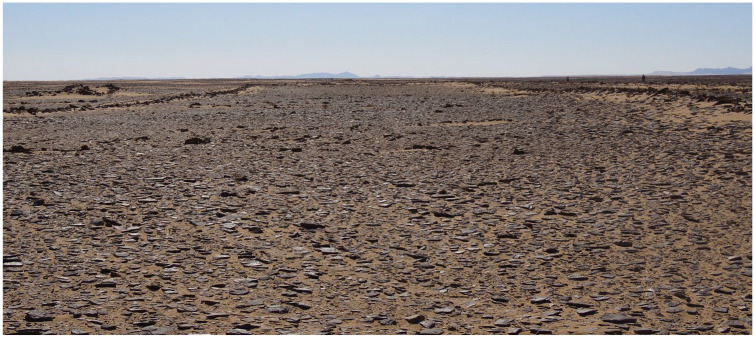
View from between walls of the largest mustatil discovered (located on right of Figure 4). Note team members on right for scale. A separate, small, mustatil is visible on the left.

**Figure 6.**
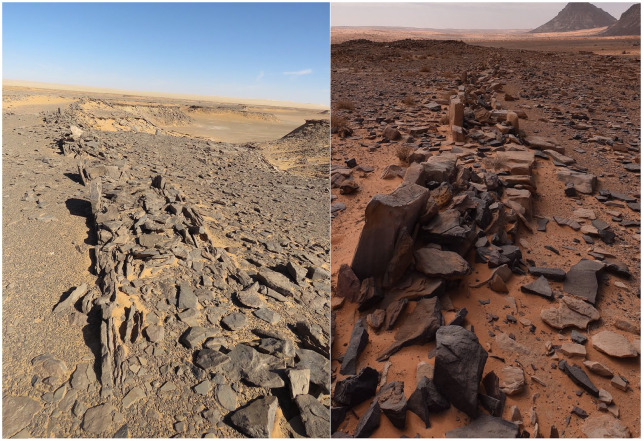
Two examples of the long walls of mustatils, both showing similar construction techniques, with outer vertically mounted tabular slabs and a rubble wall core.

Our ground survey allowed us to clarify a number of points about the architectural features of mustatils. Firstly, it is important to note that the long walls of the mustatils are always low. Even when accounting for subsequent removal of stone in some cases and wall collapse, walls seem to have always been less than half a metre high (Figures 5, 6 and Supplemental Figure S10, available online), and often less. There is likewise generally no break in the walls, a point we will return to below when considering the function of the structures. It is also clear in some cases that the walls were made by clearing the central strip of blocks: it may therefore be that this path was the goal, and the side walls somewhat ‘secondary’ to the ‘definition’ of the space between them. While not evident at all sites, the long walls at several mustatils demonstrate a similar construction method, in which vertical uprights were placed into the ground, and the gap between them filled with rubble (Figure 6).

In contrast to the diminutive long-axis walls, survey on the ground makes it clear that the two ends of the mustatil are not actually walls at all, but rather stone platforms. This is concealed by subsequent re-use in many cases, but it is clear where subsequent alteration has not changed the shape of the platforms. Examples of platforms are shown in Figure 3 and Supplemental Figure S11, available online. While partially concealed by collapse, accumulation of sand, and re-use by humans, in many cases the platforms were made with neatly faced stone, still visible in places. The platforms are often impressive in size: that at 27.387961 N, 39.379871 E, for instance is around 30 m long by 10 m in width, and over a metre high. This single example therefore contains hundreds of tonnes of rock. In many cases platforms have subsequently been used as a source of stone, typically to produce cairns (for examples see Supplemental Figure S12 available online and Guagnin et al., 2020). On the ground these can be distinguished from the original flat-topped platforms, made with layered rocks.

### Relative chronology of mustatil structures

In terms of relative chronology, our findings mirror those of Kennedy (2017) in that, based on superimposition, mustatils are consistently the oldest form of stone structure in the landscape. The most common associated younger structure are cairns (see e.g. Figure 4, Supplemental Figure S12, available online; and 27.591206 N, 40.313776 E). Other younger structures include bullseye cairns (e.g. 27.573727 N, 41.192071 E; 27.128529 N, 40.045456 E), pendant tombs (27.397202 N, 39.948100 E), keyhole tombs (27.384795 N, 39.375468 E), irregular ‘cell’ structures (26.899406 N, 39.697102 E), and stone circles (27.400836 N, 39.948904 E) (see Kennedy, 2011 for definitions of the structures used in Arabia). In 44% of the mustatils, the stone appears to have been reused exclusively to make cairns. In a further 39% of mustatils stones were reused to build a combination of structures. For instance, at 13% of the total mustatils, stones were reused to build bullseye cairns and a total of 25% of mustatils are overlain by ‘irregular structures’, lacking precise morphology but typically being characterised by irregularly shaped small ‘cells’. In total, at least 83% of mustatils in our study area have clear evidence of subsequent re-use of stone, either on top of or adjacent to mustatils. The real figure may be even higher, as satellite imagery resolution is not always high enough to tell, and in some cases sand obstructs visibility (e.g. 27.402666 N, 39.947449 E). It is also possible that future studies will indicate that some associated stone structures (such as cairns) were actually part of the mustatils rather than following after a long chronological gap.

We note that re-use of stone from mustatils is seemingly not spatially ‘random’. An indication of the changing character of landscape use is given by the selective re-use of certain mustatils, and parts of mustatils, in later periods. The parts of mustatils that are re-used to build various forms of cairns (and other features) upon them are typically also the most visible in the landscape when seen from ground-level. As shown in Figure 4 and Supplemental Figures S1 and S2, available online, cairns are frequently positioned close to the edges of scarps, making them highly visible in landscapes and forming prominent features along the skyline as seen from below the scarp. Stones from mustatils that are further from escarpments are less commonly re-used. In other words, there seems to be a change through time towards a greater emphasis upon structures being highly visible in the wider landscape.

Trying to associate this coarse relative age sequence to an absolute chronology is currently challenging. Cairns in northern Arabia often seem to date to the Bronze and Iron ages, however, Neolithic cairns are also known from the area (Guagnin 2017b, 2020). The more elaborate forms of cairn such as pendants may date to the first millennium BC, as available radiocarbon dates from these structures in Yemen vary from ca. 830 BC to 60 BC (de Maigret, 2009), but little is currently known about them. No desert kites were identified in our study area. However, we note that immediately to the east, Parr and colleagues (1978) reported the site of 205-8, north of Ha’il (which we were able to relocate: 27.739832 N, 41.551714 E). Parr and colleagues described, and illustrated, a mustatil similar to those we have discussed in this paper, both in terms of its size, shape and features such as walls made by upright slabs with rubble infill. The significance of this site, however, is that the mustatil is situated in the entrance route into a desert kite. As Parr and colleagues argue, this makes it highly likely that the mustatil is younger than the kite, as it would presumably have inhibited the kite’s functionality. Likewise, Parr and colleagues (1978) site plan suggests that stone from the kite was re-used to construct the mustatil (Supplemental Figure S13, available online). This shows that mustatils are not always the oldest stone structures in the landscape, and appear to have some temporal overlap with kites. Given that kites may have been used over thousands of years, this is perhaps not surprising. The 205-8 desert kite is a ‘star shaped’ kite, similar to those dominant in the Harrat al Sham to the north. The ‘barbed’ forms from Khaybar may be a younger phenomenon. It is also possible that mustatils were built over a long period of time. This can be tested by future research, but our impression is that the architectural homogeneity of mustatils, and their apparent concentration in only one area of Arabia, suggests that they were constructed over a limited period of time. In contrast, kites occur over a vast spatial and temporal range (e.g. Crassard et al., 2015).

### Absolute chronology

In addition to the above findings on relative chronology, we are able to present a chronometric age estimate for a mustatil for the first time, using radiocarbon dating. We recovered a piece of charcoal from a section inside a hole dug into the side of a platform at 27.385044 N, 39.338055 E by persons unknown, presumably looters (Supplemental Figure S14, available online). This platform at the southern end of a mustatil showed no evidence of reworking (e.g. into a cairn), and the sample is therefore associated with the original use of the mustatil. The charcoal was dated to 5052–4942 cal. BC (Supplemental Figure S15 and Table S2, available online), and provides a first reference of the absolute chronology of these monuments.

We also recovered a cowrie shell from a cairn (27.383524 N, 39.336853 E) 200 m south of the dated mustatil. This gave a radiocarbon age of 726–346 cal. BC (Supplemental Figure S15 and Table S2, available online). This dates to the ‘Iron Age’, and along with a ‘Bronze Age’ 2930–2770 cal. BC cairn from Jubbah (Guagnin et al., 2017b) shows later use of these landscapes, and renewed stone structure construction in post-Neolithic northern Arabia.

### Associated material culture

Overall, a striking observation is the paucity of material culture (e.g. lithics, ceramics.) associated with the mustatils surveyed. There was general excellent visibility, since they are built directly on bedrock and there is little sand cover in most cases, hence we suggest this can be taken as a genuine absence of material. Two groundstone axes were found around 27.387751 N, 39.379567 E; one in the centre of the mustatil, and one just outside. At the other end of this mustatil a low-density lithic scatter was found, centred around 27.384533 N, 39.375917 E. This consisted of non-diagnostic quartzite flakes, and was in proximity to several later structures that were built reusing stones from the mustatil, thus their age is unclear. Likewise, non-diagnostic quartz flakes were found next to the Jebel Oraf mustatil near Jubbah near a section of wall that had been reused to construct a cairn (Guagnin et al., 2020). The small number of lithics recovered offer little insight into chronology or cultural characteristics. They are generic, and neither exceedingly fresh nor highly weathered. In very broad terms they appear to be similar to the only lithics that had previously been described in broad association with a mustatil (Parr et al., 1978). In our opinion, the key observation about material culture in the landscape in and around mustatils is its paucity. An unusual artefact find comes from a mustatil at Jebel Dhaya, west of Jubbah. A fragment of a sandstone grinding stone, an artefact type common at the nearby Neolithic site of Jebel Oraf (Guagnin et al., 2020), was retouched into a scraper (Supplemental Figure S16, available online) and was found just outside the wall of the mustatil. This form of artefact seems to be rare in the region.

**Figure 7.**
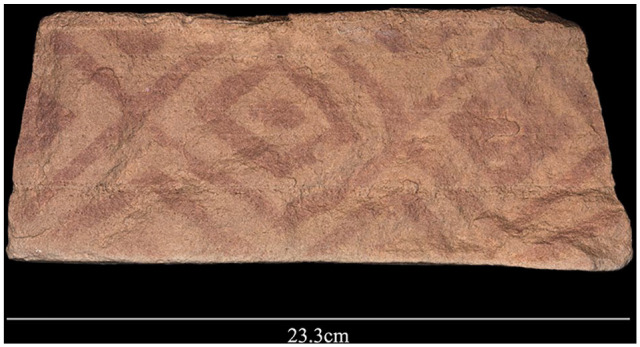
Geometric painted pattern found on a block that formed part of the platform of a mustatil.

One fascinating example of material culture, a painted rock, was found at 27.385583 N, 39.378884 E (Figure 7). The object formed part of the top course of rocks on the interior edge of the southern platform of the mustatil, and was thus part of the finished, visible surface for people to see inside the space defined by the mustatil. While paintings are known in the rock art of northern Arabia, some using pigment of a similar shade, and petroglyphs of geometric motifs have been observed in the wider area, the pattern on the rock is not currently known from other rock art contexts.

### Associated faunal remains

**Figure 8.**
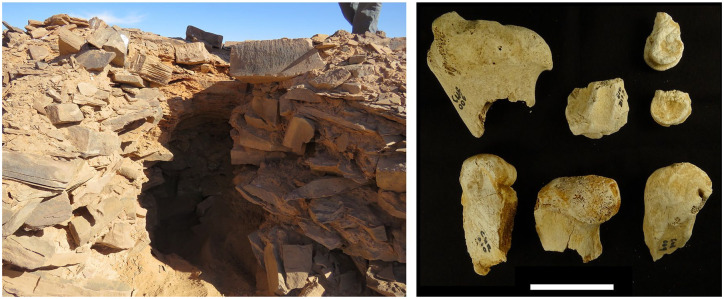
Hole dug into NEF-8 mustatil platform by persons unknown, revealing bone assemblage (right, scale = 5 cm), and leading to the recovery of charcoal from a section inside the platform.

The dated mustatil also revealed an assemblage of bones, some of which were found on the spoil heap left by the unknown diggers of the platform, and others protruding from the section. Twenty bones representing at least two taxa were recovered (Supplemental Table S3, available onlin). Two fragmented upper molars with broad U-shaped infundibulum and distinct styles (Figure 8) can be assigned to *Bos* sp. Given the timing of the site, this may well represent domesticated cattle (*Bos taurus*). However, it is also possible that they are wild aurochs (Dreschlser, 2007; Makarewicz, 2020; McCorriston and Martin, 2009; Uerpmann, 1987; Zeder, 2017). The domestication of aurochs appears to have occurred somewhere in the upper or middle Euphrates Valley at ca. 8400 BC (Helmer et al., 2005), and evidence from southern Arabia indicates the arrival or localized domestication of aurochs by at least the sixth millennium BC (McCorriston and Martin, 2009). Further studies of *Bos* fossils associated with mustatils will be required to distinguish between wild and domesticated cattle.

The remaining material consists mostly of fragmented appendicular remains (*n* = 12) that can be attributed to a smaller bovid. The remains exceed in size those of goat and sheep but are consistent in both size and morphology with *Oryx* (Supplemental Table S4, available online; cf. Peters et al., 1997). A maxilla fragment retaining a single fragmented molar is also consistent with *Oryx*, although a portion of the tooth is missing making taxonomic attribution difficult. The Arabian oryx (*O. leucoryx*) was common in the region until relatively recently, and although only known from a few prehistoric sites, its range appears to have stretched from Jordan to eastern Arabia (Uerpmann, 1987). As the only suitably sized bovid known from the region (but see Harrison and Bates, 1991 and Guagnin et al., 2018), we tentatively assign the remains to this endemic species. The recovery of two right distal femur fragments and a juvenile (unfused) distal femur epiphysis, as well as evidence for *Bos*, indicate the presence of at least four individual bovids at the site. Importantly, this indicates the presence of hunted fauna, and possibly domesticated fauna in the case of *Bos*, buried at the site.

Fossils are poorly preserved with most exhibiting weathering cracks (*n* = 15, 75%) and bleaching (*n*=15, 75%). The latter is likely the result of insolation and abrasion by fine wind-blown sand, processes that are particularly prevalent in desert settings (Ferández-Jalvo and Andrews, 2016). A number of unweathered (*n* = 5, 25%) and unbleached (*n* = 5, 25%) specimens indicate that weathering took place following the exhumation of the remains. No additional bone surface modifications (e.g. butchery marks) were identified, although it is possible that weathering has removed/obfuscated these.

## Discussion

The archaeological record of northern Arabia indicates that a remarkable development had occurred by around 5000 BC. The creation of monumental landscapes featuring hundreds of large-scale stone monuments represents a significant cultural change, and a transformation of the landscape.

The function of mustatils remains enigmatic, though based on our combined satellite and field findings, some possibilities can be highlighted and explored by future research. A number of features evident on the ground, and not visible on satellite imagery, furnish crucial information on mustatils. For instance, the long walls are very low and typically lack obvious entry points, and therefore do not seem to be obviously functional as something like animal corrals. Likewise, the morphology and landscape position of these structures argue against other ‘pragmatic’ possibilities such as water storage. While mustatils are often located near prominent landscape features such as lakes and sandstone jebels, they do not seem to emphasise being highly visible in the landscape, in contrast to many (but not all) later structures, such as different forms of cairns/tombs. What becomes clear on the ground is that structures are primarily defined in structural terms by large stone platforms at either end, with the low walls between them denoting a perhaps conceptually, rather than effectively, enclosed space. The discovery of a painted rock on the interior aspect of a mustatil may also provide an indication of the ritual function of these structures. This painted, and faced, slab shows that the interior face of the platforms was sometimes decorated, indicating a consideration of the presence of viewers who were inside the space created by the platforms and the walls. This is consistent with the space defined by mustatils being localities for ritual behaviours.

As well as their locations and architectural features, an important clue to the functioning of mustatils comes from the general paucity of associated material culture. This observation indicates that these structures were not sites of long-term, repeated, habitation. The absence of hearths and lithics stands in stark contrast to contemporaneous sites in the region (Scerri et al., 2018b; Guagnin et al. 2020). Likewise, the frequent building of mustatils in close proximity to each other also suggests that an important aspect of their use concerns the act of building the structures, rather than their actual long-term use. The seeming redundancy in mustatil construction suggests an emphasis on community cooperation as a key aspect of understanding their function. We can therefore consider two forms of ritual in relation to mustatils, one of ritual behaviours carried out within them, and another of the actual process of their construction. All of this contrasts with desert kites, that are argued to have an essentially practical function (although of course one which also had significant social and economic ramifications). The lack of obvious utilitarian functions for mustatils suggests a ritual interpretation. In fact, mustatils seemingly represent one of the earliest examples known anywhere of large-scale ritual behaviours encoded in the practice of monumental construction and use.

The wider regional context aids consideration of the function and significance of mustatils. While no exact equivalent exists and the unique character of mustatils must be emphasised, there may be some form of relationship between mustatils and two forms of structure in the southern Levant. Firstly, there are the rectangular platforms associated with late Neolithic cairns, as previously mentioned at the start of this paper (Abu-Azizeh et al., 2014). Secondly, ‘cultic’ sites, often described as shrines and sanctuaries, have been recorded across the arid southern Levant (e.g. Avner, 1984; Eddy and Wendorf, 1999; Fujii, 2013, 2014; Rosen, 2015). These are varied, but often consist of rectangular structures with a ritual/monumental character. While poorly dated they cover a similar period to that associated with the mustatil phenomenon. Indeed Rosen (2008: 121) describes a ‘virtual explosion in desert cult’ in the later part of the sixth millennium BC, the centuries immediately preceding the building of the mustatil we have dated. These cultic sites are not identical to mustatils – they are smaller, they lack platforms, and they are generally more internally subdivided. Yet the presence of rectangular structures with a ritual character in the area from which populations and ideas may have spread into Arabia does suggest one possible inspiration for mustatils. Like the mustatils, the southern Levantine shrines also are also characterised by a sparsity of associated material culture (e.g. Fujii, 2014; Rosen, 2015). In wider terms, recent archaeological research in Jordan has also identified the expansion of human groups into new areas on the margins of the Levant in the 7^th^ and 6^th^ millennium BC and the development of nomadic pastoralism (e.g. Fujii, 2013; Rollefson et al., 2014; Rowan et al., 2015). This provides important context for developments further south, on the Arabian Peninsula. Just as we have proposed that the mustatils phenomenon reflects the emergence of territorial behaviours in northern Arabian pastoral communities, so ‘cultic sanctuaries’ in the southern Levant have been seen as reflecting territoriality linked to the emergence of fully nomadic pastoralism (Rosen, 2008).

While there are hints of similarities to previously known structures from the Levant, the cultural background of the mustatils and the details of timings and dynamics of any population movements and cultural influences remain poorly understood. The mustatil phenomenon represents a specific development within Arabia. Our emphasis on the importance of the platforms in mustatils suggest a possible connection to southern Arabia with sites featuring platforms such as the ca. 4400 BC site of Shi’b Kheshiya (McCorriston et al., 2011). In fact, the dated mustatil at ca. 5000 BC falls both spatially and temporally between the sixth millennium BC rectangular structures of the Levant and the fifth millennium BC platform structures of southern Arabia, as discussed above. How the recently dated Dûmat al-Jandal stone platform (Munoz et al., 2020), originating in the sixth millennium BC, relates to mustatils remains to be clarified. The southern Arabian sites are interpreted as locations where social aggregation occurred, in the context of emerging territoriality, perhaps in the form of ritual slaughter and/or conspicuous consumption (cf e.g. Henton et al., 2014). We suggest that mustatils probably played a similar role, and represent one manifestation of the increasing territoriality that developed, induced by factors such as competition for grazing land in the challenging and unpredictable environments of Arabia. Even during the wettest time of the Holocene humid period, the environment would have been highly seasonal and droughts would have occurred (Guagnin et al., 2016). The presence of bones of wild fauna (a medium sized bovid, attributed to *Oryx*) and either domesticated cattle or wild auroch inside a platform of a mustatil is consistent with this notion of some form of community gathering. The presence of wild fauna is interesting given the late Neolithic age of the site, and may variably be interpreted as indicating a mixed economy of pastoralism and hunting, a ‘return’ to a hunter-gatherer lifestyle, or a ritual aspect (for an analogous example of the latter, see Bar-Oz et al., 2011). The burial of fauna in monuments also shares some similarity with the ‘cattle cult’ of the Sahara, where from about 4440 BC cattle burials are found within megalithic stone structures (Di Lernia, 2006).

McCorriston and colleagues (2011: 18) suggested that the platform structures of southern Arabia marked ‘the first sign that people were constructing larger-scale social group identities’. They suggest that this ‘signals a strengthened collective identity linked perhaps to maintaining and defending access to strategic resources’ such as pasture and water. We suggest a similar explanation applies in northern Arabia, at an earlier date. Whether there was a continuous developmental sequence from north to south in Arabia, or convergent evolution in similar circumstances, remains to be seen. This, and the precise environmental conditions in which mustatils emerged, should be key foci for future studies involving the excavation of mustatils. Our findings indicate that mustatils, and particularly their platforms, are significant archives of Arabian prehistory, and their future investigation and excavation is likely to be highly rewarding, leading to a better understanding of social and cultural developments.

## Supplemental Material

Groucutt_et_al_SI_revised_without_track_changes – Supplemental material for Monumental landscapes of the Holocene humid period in Northern Arabia: The mustatil phenomenon Click here for additional data file. Supplemental material, Groucutt_et_al_SI_revised_without_track_changes for Monumental landscapes of the Holocene humid period in Northern Arabia: The mustatil phenomenon by Huw S Groucutt, Paul S Breeze, Maria Guagnin, Mathew Stewart, Nick Drake, Ceri Shipton, Badr Zahrani, Abdulaziz Al Omarfi, Abdullah M Alsharekh and Michael D Petraglia in The Holocene
