# Supplementary material for: Monumental landscapes of the Holocene humid period in Northern Arabia: The mustatil phenomenon
Source: Holocene. 2020 Aug 17;30(12):1767–79. doi: 10.1177/0959683620950449 (PMC7575307; doi:10.1177/0959683620950449)
Supplement: Groucutt_et_al_SI_revised_without_track_changes – Supplemental material for Monumental landscapes of the Holocene humid period in Northern Arabia: The mustatil phenomenon [file Groucutt_et_al_SI_revised_without_track_changes.pdf]

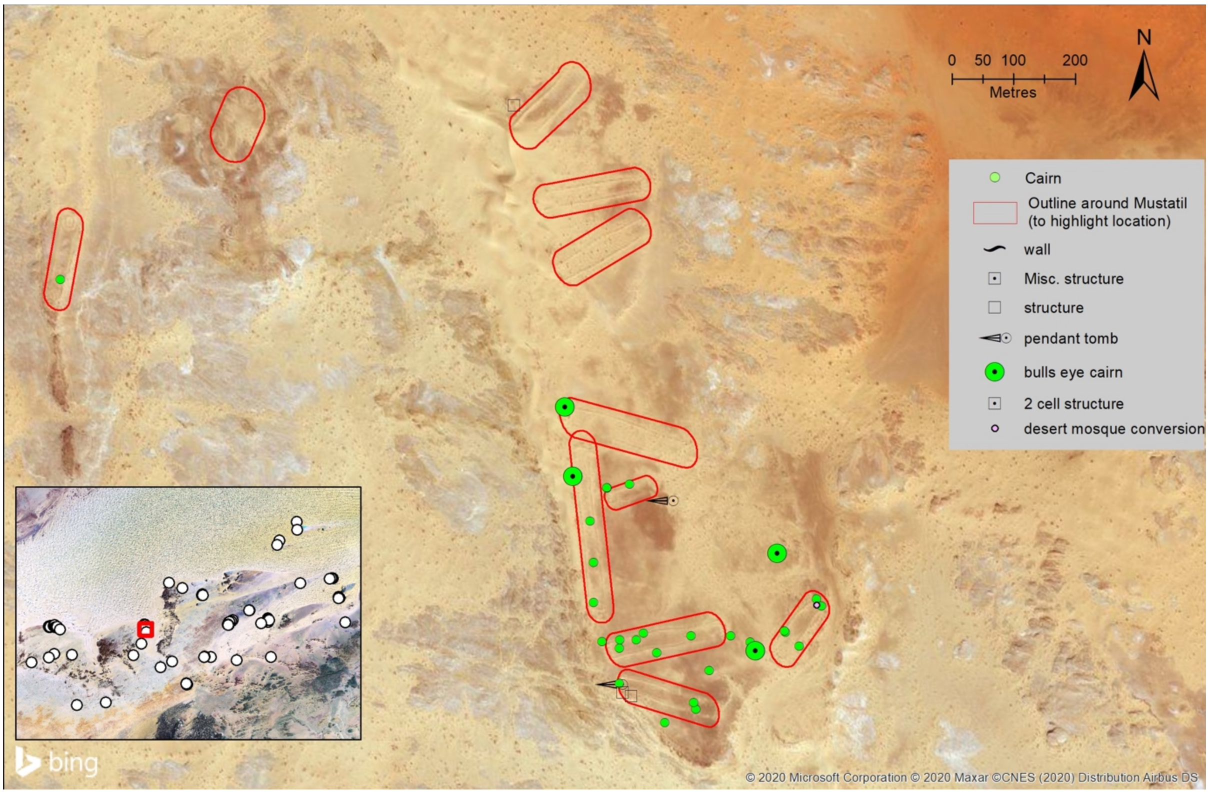

Figure S1 – A group of mustatils (27.3962 N, 39.9478 E), with prominent examples of subsequent (younger) structures labelled.

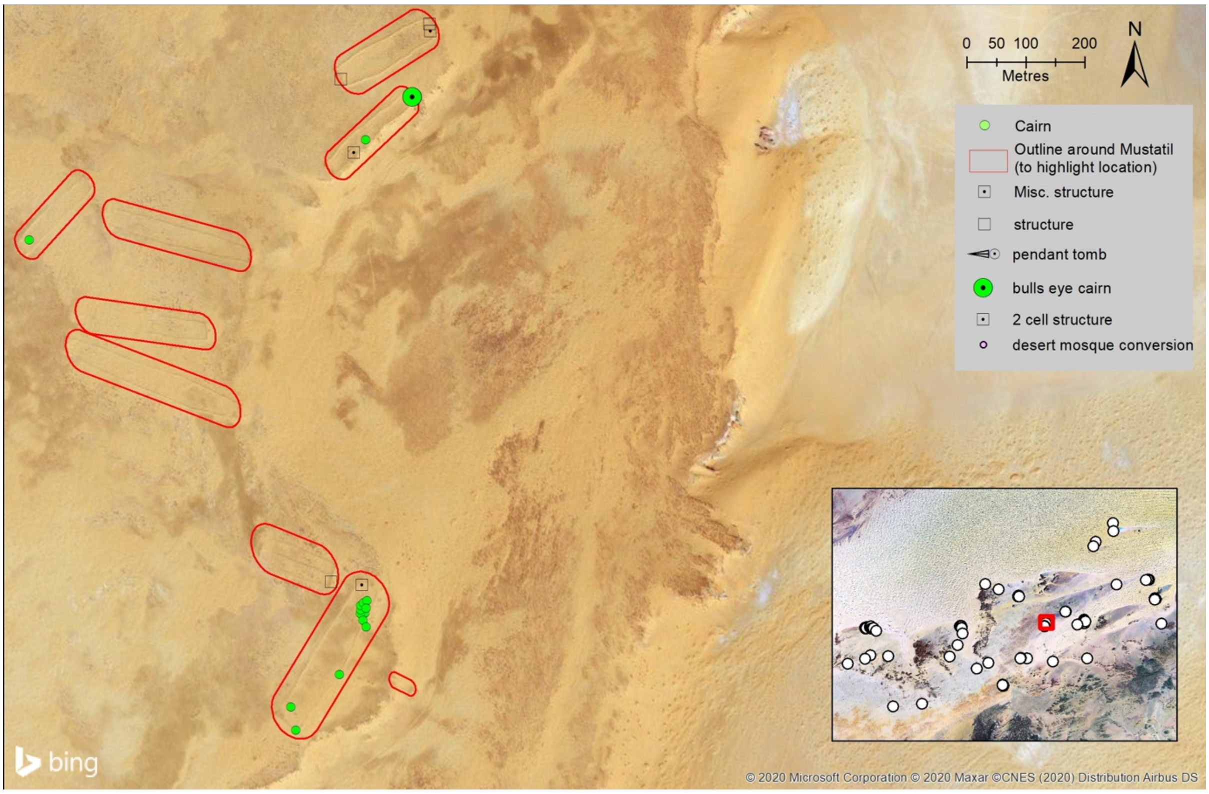

Figure S2 – A group of mustatils (27.4160 N, 40.4882 E). The area to the east is a low-lying area which would have held water during wetter periods.

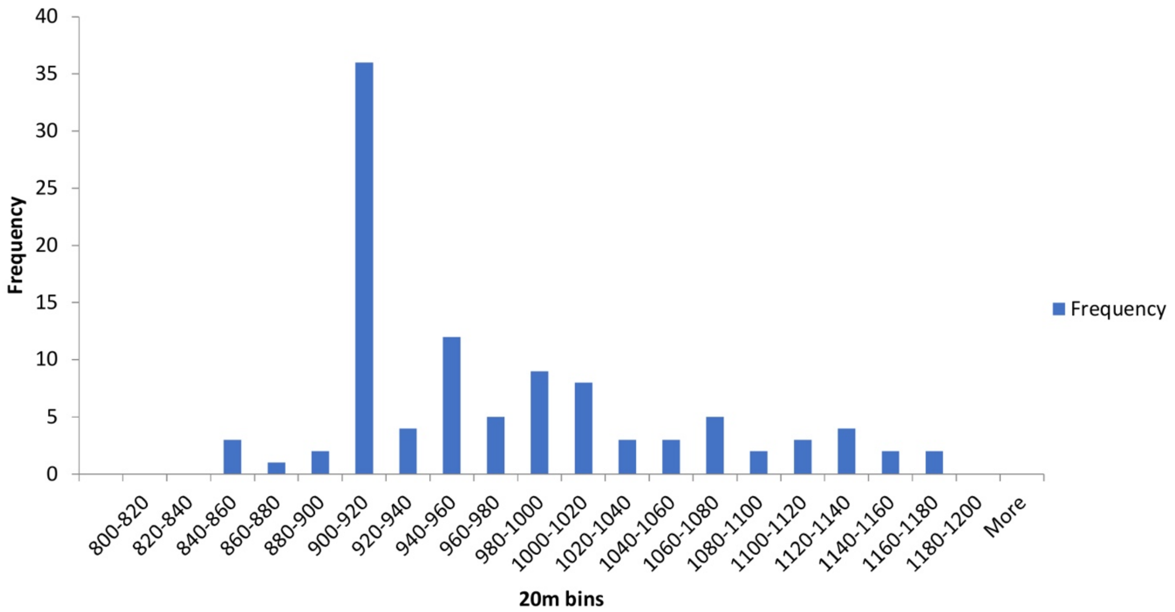

Figure S3. The altitude of mustatil locations.

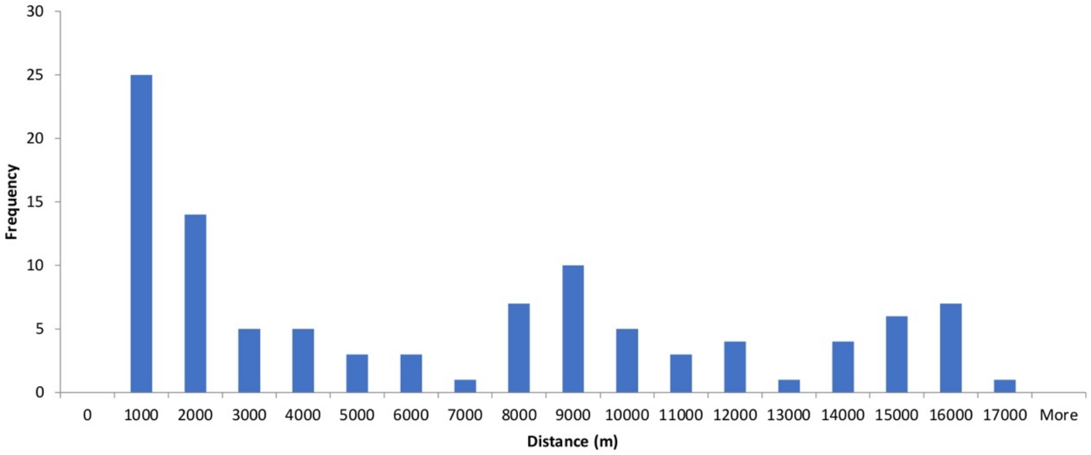

Figure S4. The distance of mustatils to nearest palaeolake deposits.

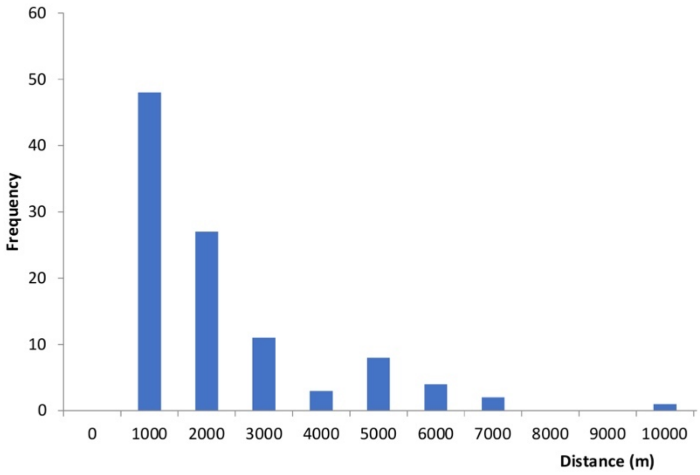

Figure S5. The distance of mustatils to nearest past water source (inc. 50km rivers outside dunes).

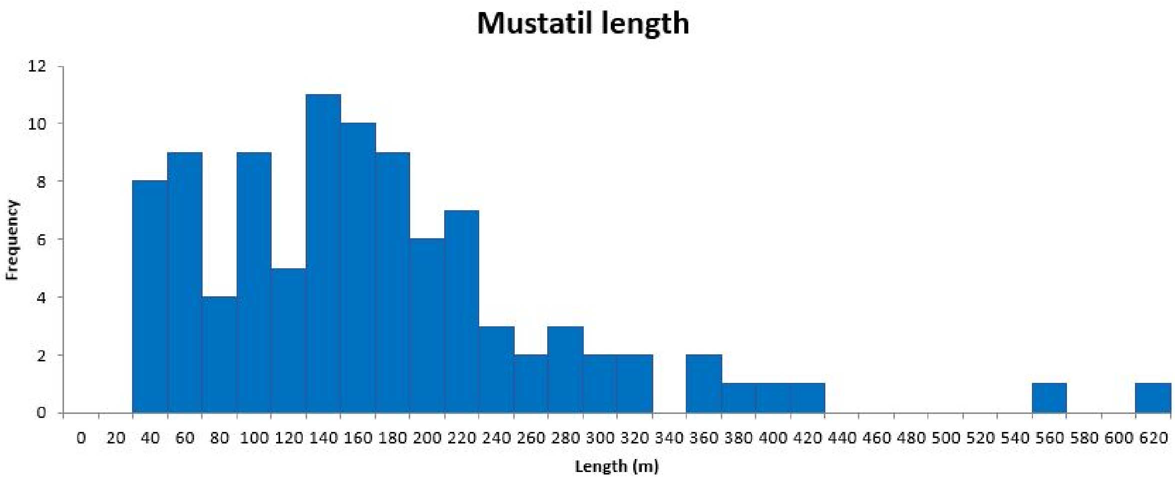

Figure S6. The length of mustatils (metres).

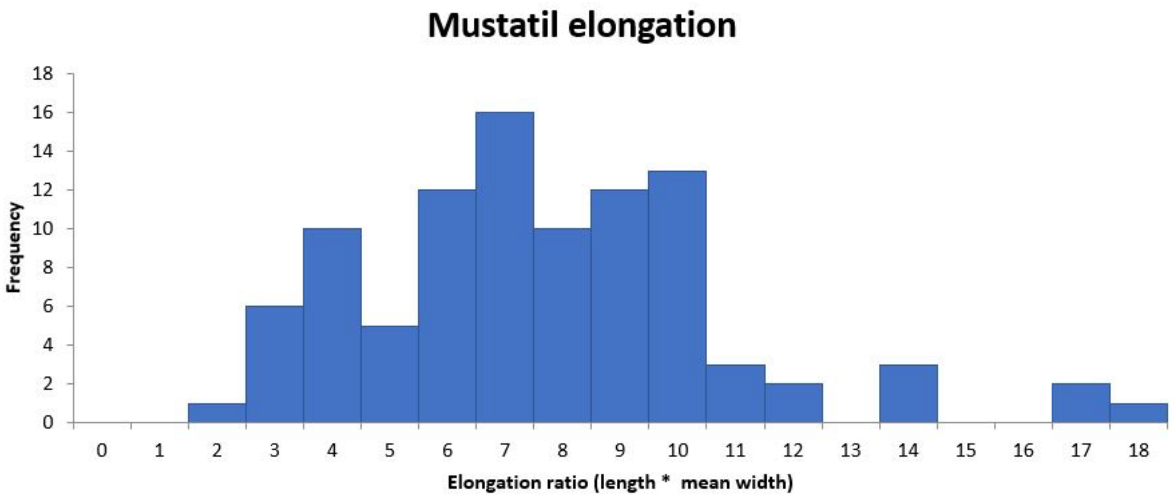

Figure S7. The elongation of mustatils.

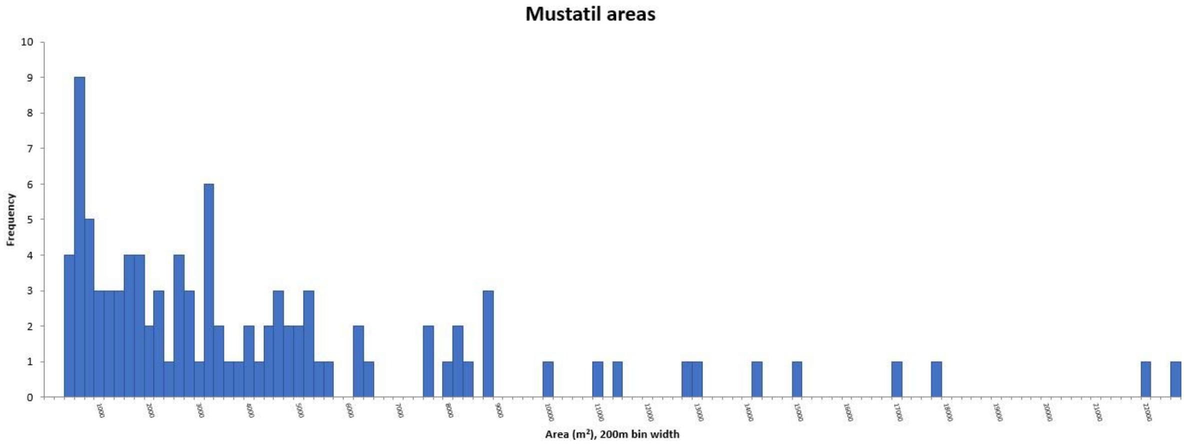

Figure S8. The area (m^2) of mustatils.

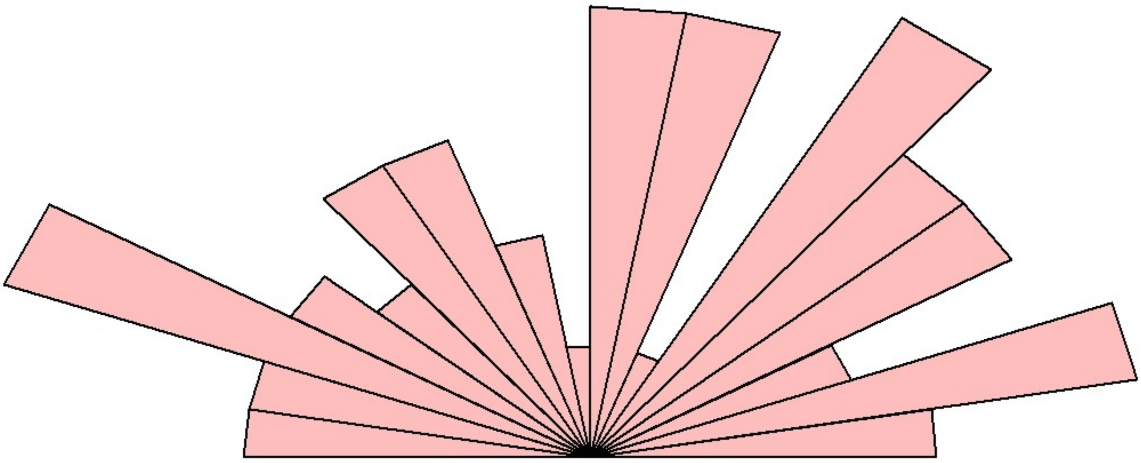

Figure S9 – Mustatil orientations. Shown in 10 degree increments, mirrored south of the E-W axis. As PRS' are linear, the rose histogram is mirrored through its centre, thus only half is shown here. The length of each segment indicates the number of mustatils sharing this axis of orientation.

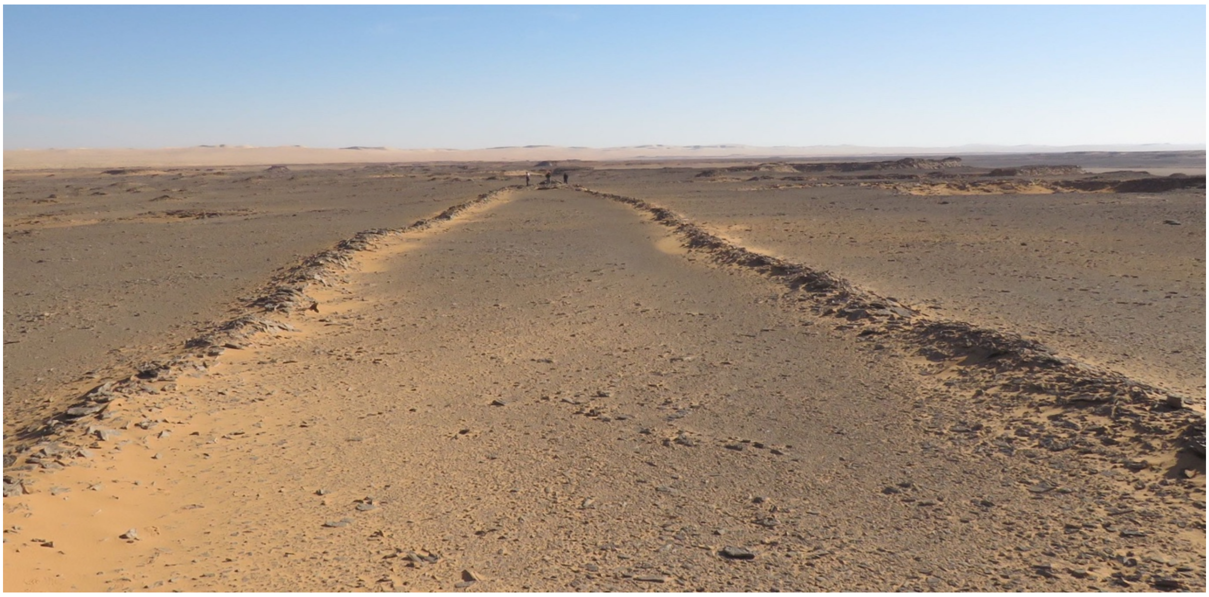

Figure S10 – Example of typical low walls of a mustatil.

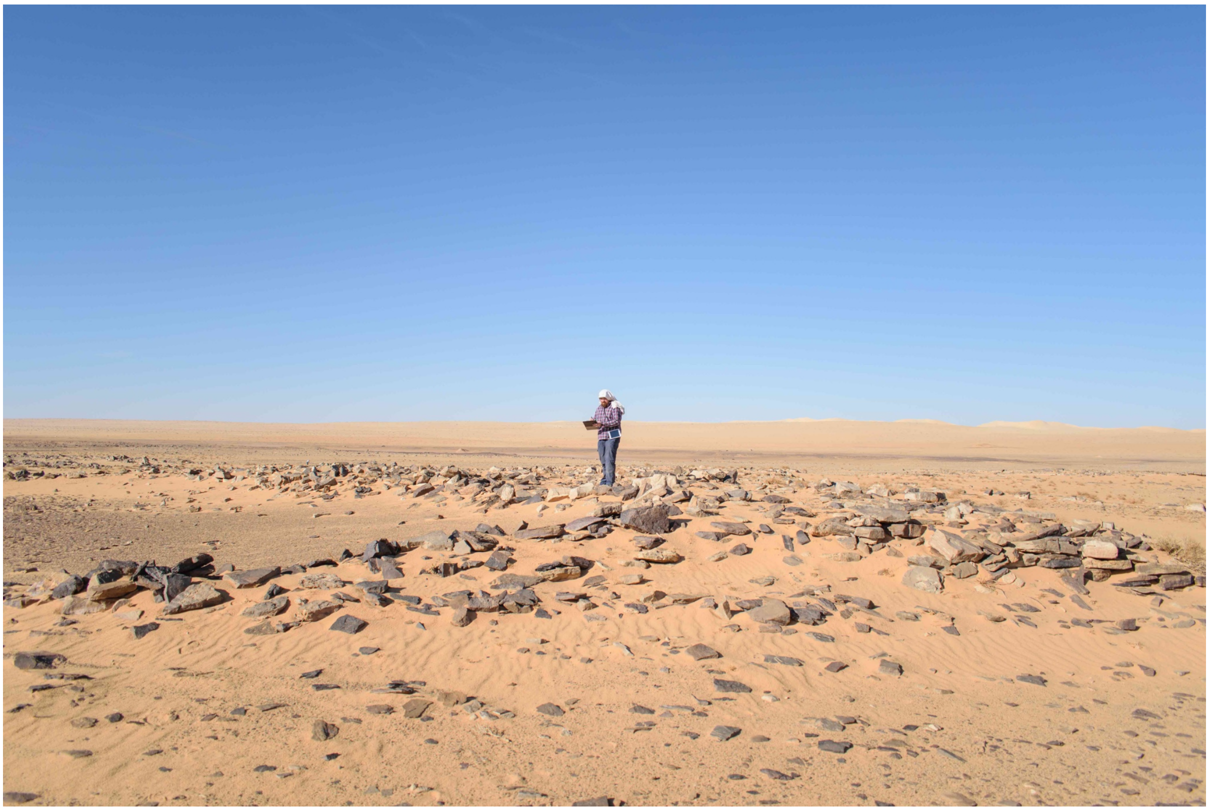

Figure S11 – Example of a platform at the end of a mustatil.

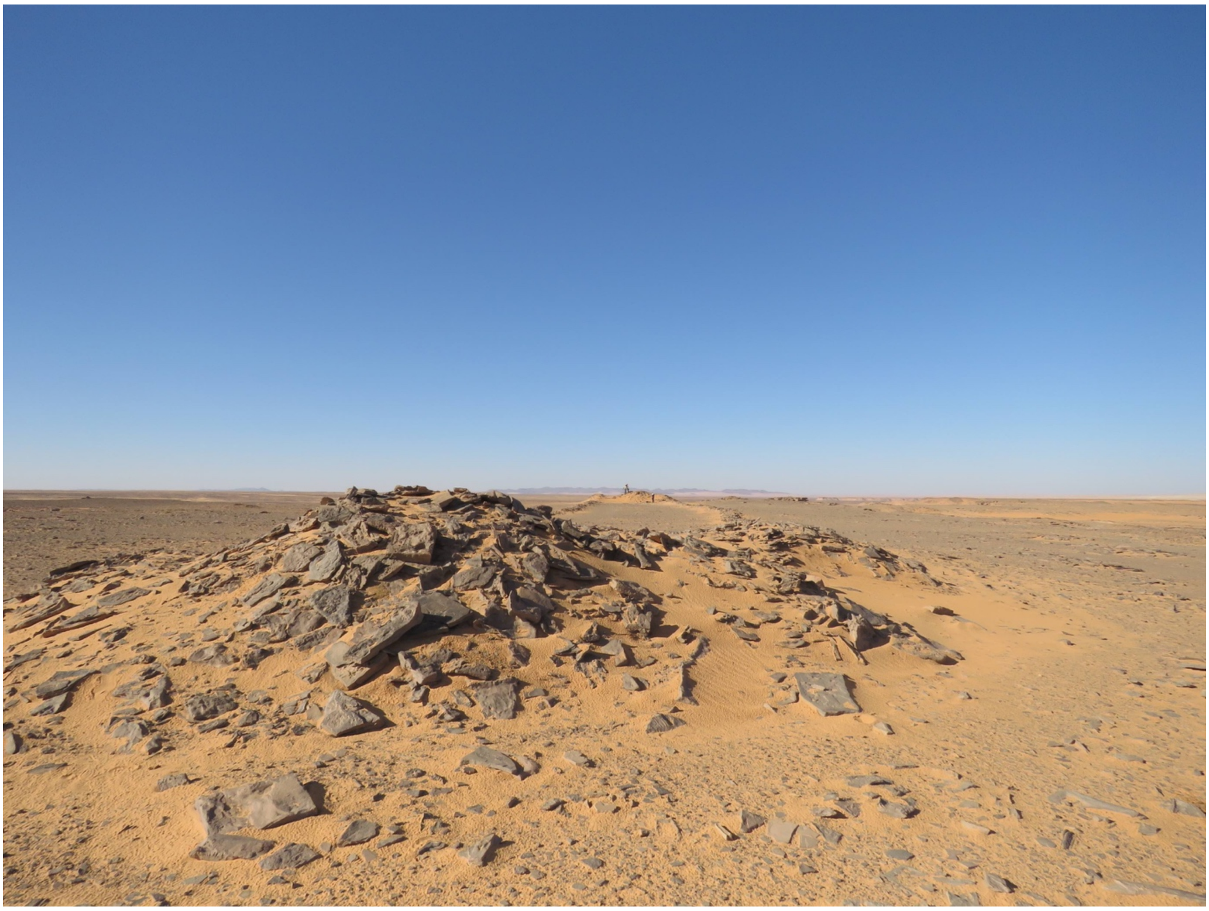

Figure S12 – Example of a mustatil where platforms at both ends have been modified by subsequent construction of cairns.

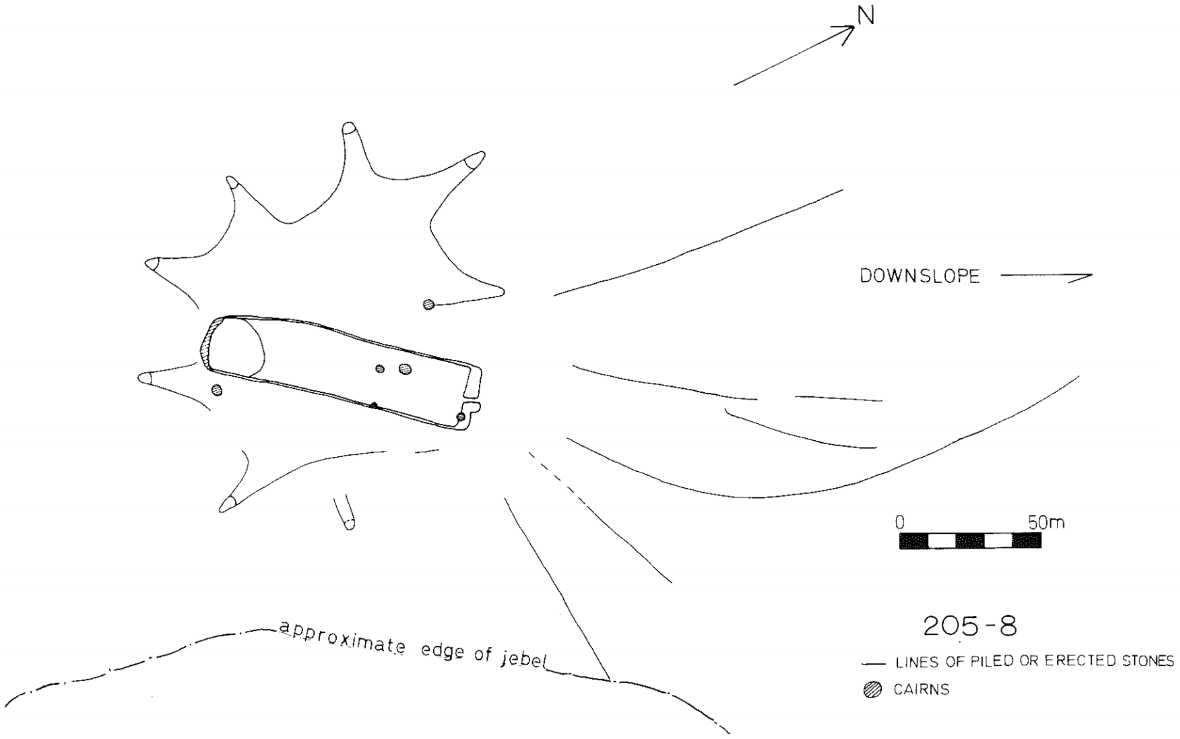

Figure S13 – 205-8 site plan (after Parr et al., 1978).

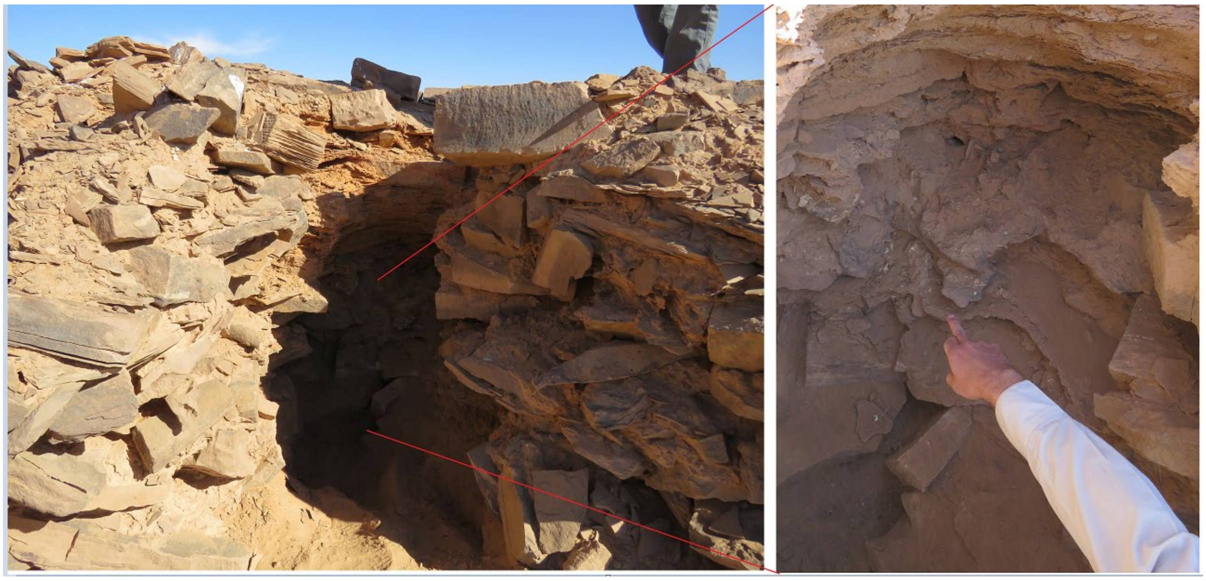

Figure S14. Hole made by persons unknown into platform of mustatil. Bones were found spread down slope (behind camera). Right shows close up of section, where bone fragments can be seen in situ. Charcoal was recovered where finger is pointing.

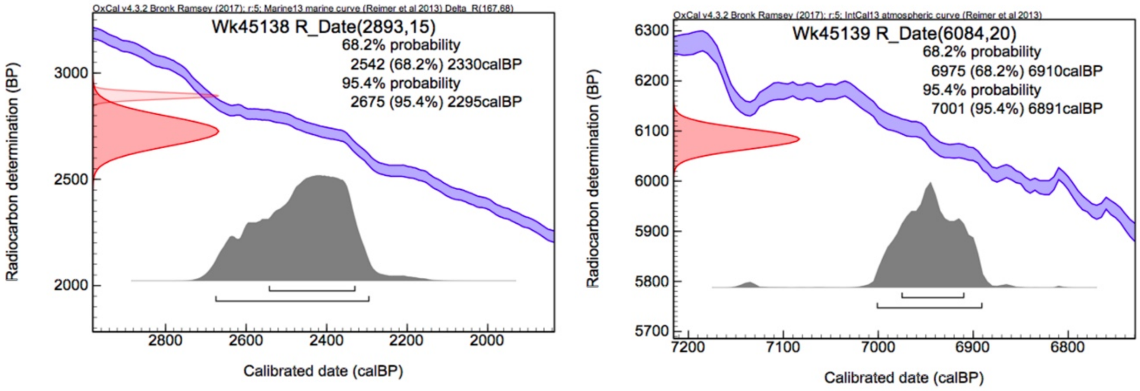

Figure S15. Radiocarbon results for cairn (left) and mustatil (right).

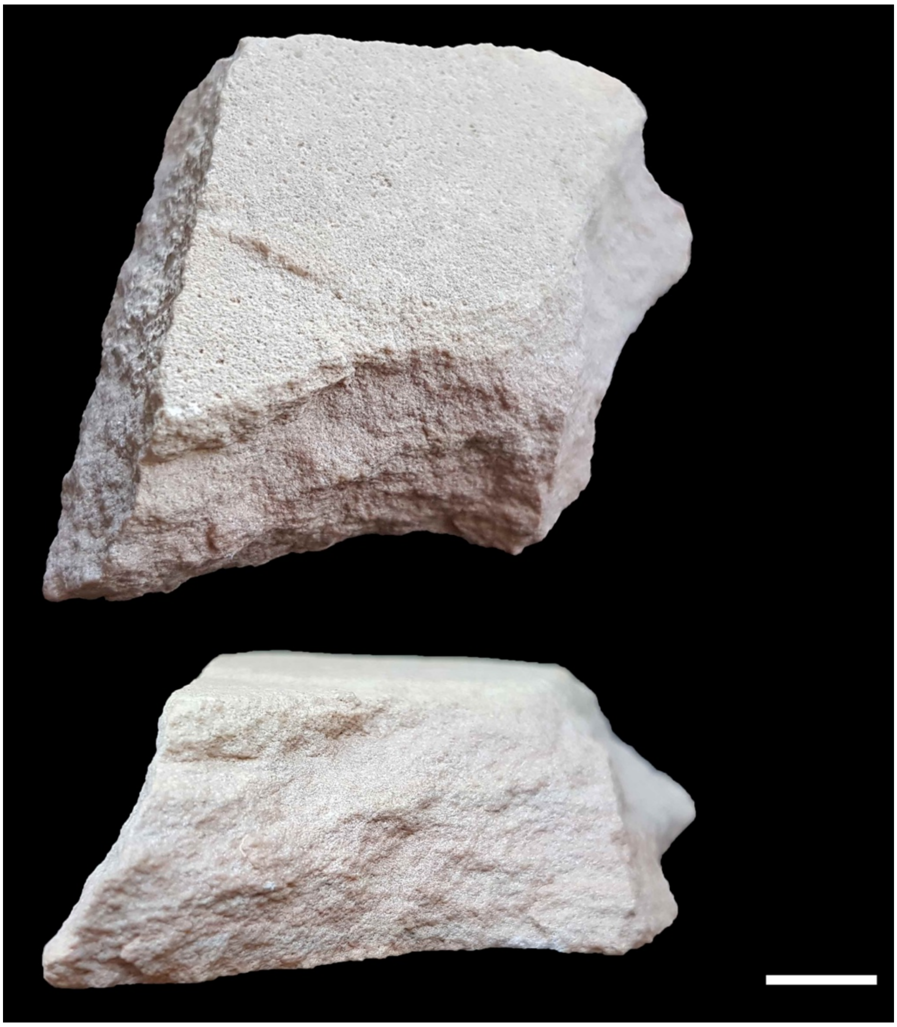

Figure S16. Sandstone scraper from beside mustatil at Jebel Dhaya, Jubbah. Scale = 1cm.

Tables

Table S1. Data on mustatil dimensions. Length = measured between middle points of outside edges of platform at either end, width = average width, elongation = length/width, area = measured polygon. NB, length could not always be measured due to subsequent modification of structure.

	Length (m)	Width (m)	Elongation	Area (m^2)
**N.**	97	104	97	97
**Min.**	26.0	7.0	1.9	259
**Max.**	616.0	79.0	17.9	22,558
**25th %**	84.5	14.4	5.1	1,243
**75th %**	204	26.9	9.0	5,198
**Mean.**	161.1	21.7	7.2	4,363
**Median.**	142.0	20.8	6.8	2950
**Std Dev.**	107.0	9.9	3.1	459

Table S2. Radiocarbon results.

Sample ID	Material and context	D^{14C}	$F^{14}C\%$	Result (uncal.)
WK45138	Cowrie shell from cairn at 27.383517 N, 39.336863 E	-302.4 ± 1.4 ‰	69.8 ± 0.1	2893 ± 15 BP
WK45139	Charcoal from section inside southern platform of mustatil at 27.385036 N, 39.338050 E.	-531.1 ± 1.2 ‰	46.9 ± 0.1	6084 ± 20 BP

Table S3. Faunal list.

Specimen	Taxon	Element	Side	Portion

001	cf. *O. leucoryx*	Tibia	Left	Proximal epiphysis + shaft
002	Bovidae	Crania	Indet.	Frontal
003	Bovidae	Tooth	Indet.	Upper molar
004	cf. *O. leucoryx*	Radius	Right	Proximal epiphysis + metaphysis
005	*Bos* sp.	Tooth	Indet.	Infundibulum
006	cf. *O. leucoryx*	Humerus	Right	Proximal epiphysis + metaphysis
007	cf. *O. leucoryx*	Humerus	Right	Distal epiphysis + metaphysis
008	cf. *O. leucoryx*	Femur	Right	Proximal head + neck
009	cf. *O. leucoryx*	Femur	Right	Greater trochanter
010	cf. *O. leucoryx*	Femur	Left	Distal epiphysis + metaphysis
011	cf. *O. leucoryx*	Femur	Left	Distal epiphysis + metaphysis
012	cf. *O. leucoryx*	Femur	Indet.	Distal epiphysis
013	cf. *O. leucoryx*	Radius	Left	Distal epiphysis + metaphysis
014	Indeterminate	Rib	Indet.	Articular end
015	cf. *O. leucoryx*	Metapodial	Indet.	Distal epiphysis (1/2)
016	cf. *O. leucoryx*	Metapodial	Indet.	Distal epiphysis (1/2)
018	cf. *O. leucoryx*	Vertebra	NA	Centrum (unfused)
019	*Bos* sp.	Tooth	Indet.	Upper molar
020	Bovidae	Crania	NA	Pharyngeal tubercle
021	cf. *O. leucoryx*	Maxilla	Left	Alveolar process

Table S4. Measurements of various bovid limb bones and recent comparative alcelaphine, hippotragine, and caprine material.

Specimen / species	Humerus BT	Humerus Bd	Femur DC	Tibia Bp
007	43.0	46.0	-	-
008	-	-	30.5	-
009	-	-	-	60.0
Alcelaphus buselaphus [γ]	47.5–57.5<br>(*n* = 4)	53.0–62.0<br>(*n* = 4)	32.0–40.0<br>(*n* = 4)	67.0–82.0<br>(*n* = 3)
Oryx dammah [γ]	45.0–49.5<br>(*n* = 12)	49.0–55.5<br>(*n* = 12)	30.5–33.0<br>(*n* = 12)	61.0–70.0<br>(*n* = 12)
Oryx gazella [γ]	50.5–59.0<br>(*n* = 8)	51.5–66.5<br>(*n* = 8)	35.5–42.5<br>(*n* = 8)	75.0–89.0<br>(*n* = 8)
Oryx beisa [γ]	45.5–53.0<br>(*n* = 6)	51.0–59.0<br>(*n* = 6)	31.5–37.5<br>(*n* = 6)	67.0–77.0<br>(*n* = 6)
Oryx leucoryx [γ]	40.0–41.5<br>(*n* = 3)	43.5–46.5<br>(*n* = 3)	25.0–26.5<br>(*n* = 3)	53.0–56.0<br>(*n* = 3)
Capra hircus	27.7–40.8 [α]<br>(*n* = 42)	27.0–44.6 [α]<br>(*n* = 42)	21 ± 2 [β]<br>(*n* = 52)	-
Ovis aries	23.1–35.0 [α]<br>(*n* = 56)	23.9–37.0 [α]<br>(*n* = 56)	24 ± 2 [β]<br>(*n* = 46)	-

* Measurements taken following von den Driesch (1976).
[γ] Data from Peters et al. (1997)
[α] Data from Salvango & Albarella (2017)
[β] Data from Zedda et al. (2017)

Supplementary References

Peters, J, van Neer, W, Plug, I, (1997) Comparative postcranial osteology of hartebeest (Alcelaphus buselaphus), scimitar oryx (Oryx dammah) and addax (Addax nasomaculatus),

with notes on the osteometry of gemsbok (Oryx gazelle) and Arabian oryx (Oryx leucoryx). Annalen Zoologische Wetenschappen 280: 1–83.

Salvango, L, Albarella, U, (2017). A morphometric system to distinguish sheep and goat postcranial bones. PLoS ONE 12: e0178543.

Von den Driesch, A (1976) A Guide to the Measurement of Animal Bones from Archaeological Sites. Peabody Museum, Harvard University.

Zedda, M, Palombo MR, Brits D, et al. (2017) Differences in femoral morphology between sheep (Ovis aries) and goat (Capra hircus): macroscopic and microscopic observations. Zoomorphology 136: 145–158.
